# Case report: meningitis as a presenting feature of anti-NMDA receptor encephalitis

**DOI:** 10.1186/s12879-020-4761-1

**Published:** 2020-01-07

**Authors:** Maria Stavrou, Jing Ming Yeo, Alexander David Slater, Oliver Koch, Sarosh Irani, Peter Foley

**Affiliations:** 1 Centre of Clinical Brain Sciences, The University of Edinburgh, Chancellor’s Building, 49 Little France Crescent, Edinburgh, EH16 4SB UK; 2 Anne Rowling Regenerative Neurology Clinic, 49 Little France Crescent, Edinburgh, EH16 4SB UK; 3 Department of Clinical Neurosciences, Western General Hospital, Edinburgh, EH4 2XU UK; 4 Neurology Department, Queen’s Medical Centre, Nottingham, NG7 2UH UK; 5 Medical Education Centre, Monklands Hospital, Airdrie, ML6 0JS UK; 6 Regional Infectious Disease Unit, Western General Hospital, Edinburgh, EH4 2XU UK; 7 Nuffield Department of Clinical Neurosciences, University of Oxford, Oxford, OX3 9DU UK

**Keywords:** Encephalitis, Meningitis, Anti-NMDA receptor, Anti-NMDAR

## Abstract

**Background:**

Meningitis is a very rare atypical presenting feature of anti-NMDA receptor encephalitis. In our case report, we describe an unusual clinical presentation of anti-NMDA receptor encephalitis with a biphasic pattern of meningitis followed by encephalitis and discuss potential mechanisms underlying this presentation. We aim to widen the differential diagnosis to be considered in a patient presenting with clinical meningitis and pyrexia.

**Case presentation:**

This is a case of a 33-year old Caucasian woman who initially presented with a lymphocytic meningitis attributed to a viral infection. She subsequently developed fluctuating consciousness, agitation, visual hallucinations, dyskinetic movements, a generalized tonic-clonic seizure, and autonomic instability. Investigations revealed a diagnosis of anti-NMDA receptor encephalitis secondary to a previously unidentified ovarian teratoma. She made an excellent recovery with immunotherapy and removal of the teratoma.

**Conclusion:**

Clinicians should consider autoimmune encephalitides in individuals with meningitis, particularly where extensive investigations fail to identify a causative pathogen and there is rapid development of an encephalitic phenotype.

## Background

Anti-NMDA receptor encephalitis is a well-recognised immunotherapy-responsive condition which often occurs as a paraneoplastic phenomenon. A typical presentation is in a young individual with a viral-like prodrome followed by the development of severe psychiatric symptoms, memory loss, seizures, reduced consciousness and sometimes orofacial dyskinesias, and progression to autonomic and respiratory instability. Meningitis is a very rare presenting feature of anti-NMDA receptor encephalitis with our literature search only revealing one other reported case. We present a case of a 33-year old Caucasian woman who initially presented with a lymphocytic meningitis attributed to a viral infection, but who subsequently developed clinical and investigative features consistent with anti-NMDA receptor encephalitis. The aim of this report is to present and discuss possible mechanisms underlying this atypical presentation.

## Case presentation

A 33-year old, previously fit and well, Caucasian woman presented to a UK acute medical unit with 2 days of gradual onset pressure-like headache, fever, neck stiffness and vomiting. There was no travel history of note or recent ill contacts. On examination, she was febrile at 38.6 °C and had photophobia with nuchal rigidity, however with a normal conscious level, and unremarkable systemic and neurological examinations. Initial tests included a normal serum white cell count and a C-reactive protein of < 1 mg/L, normal creatinine and electrolyte levels, and a normal chest radiograph. A Computed Tomography (CT) of her brain was reported as normal with no masses, haemorrhage or hydrocephalus. Cerebrospinal fluid (CSF) analysis revealed an elevated white cell count of 172 × 10^6^/L, with complete lymphocytosis and elevated CSF pressure of 32 cm H_2_0 (Table 1). A working diagnosis of viral meningitis was made. Following a lumbar puncture, she was treated empirically with intravenous acyclovir 10 mg/kg three times a day pending her CSF virology polymerase chain reaction (PCR) screen result which came back negative after 3 days at which point the acyclovir was stopped (Table 1). She also received paracetamol as an analgesic and antipyretic but did not receive steroids. Her headache improved, she was afebrile for 48 h and she was discharged home after 5 days.

**Table 1:** Summary of serum and cerebrospinal fluid investigations

SERUM			CEREBROSPINAL FLUID (Three lumbar punctures performed on admission days 1, 13 & 23) (reference range)			
Infective	Autoimmune	Tumour markers (reference range)				
			Admission day	1	13	23
Negative for: HIV antigen/antibody Hepatitis C antibody Anti-treponemal IgG CMV IgM and IgG EBV IgM Leptospirosis IgM Borrelia antibody Cryptococcal antigen Blood cultures	Negative for: Anti-VGKC MPO & PR3-ANCA C3 and C4 ANA Anti-CCP Anti-Ro Anti-La Anti-Smith Anti-RNP Anti-Scl-70 Anti-Jo	LDH: 265 (125–220 units/liter) CA-125: 8 (0–35 units/mL) β-HCG: < 1 (< 5 m-international units/mL) AFP: < 2 (< 6 ng/mL) No paraproteins Normal immunoglobulin levels.	Opening pressure (cm H_2_0) (5–20)	32	17	Not available
			White cell count (× 10^6^/L) (< 5)	172 (100% lymphocytes)	85 (Differential not available)	143 (100% lymphocytes)
			Red cell count (×10^6^/L) (0)	35	6	0
			Protein (g/L) (0.15–0.60)	0.77	1.07	0.32
			Glucose(serum glucose) (g/L) (60–80% of serum)	2.3 (no serum)	2.5 (10·5)	3.8 (6·1)
			Cryptococcal antigen	–	Negative	–
			ANCA	–	Negative	–
			Microscopy	No organisms seen (all three samples)		
			Microbiology (by PCR)	Negative for: Bacteria: H. influenza, *S. pneumoniae*, N. meningitidis (all samples) Virus: enterovirus, HSV 1 & 2, VZV, parechovirus (all samples)		
			Culture and sensitivity	No growth, No mycobacteria (all samples)		
			Cytology	No malignant cells detected (all samples)		

*HIV* Human Immunodeficiency Virus, *CMV* Cytomegalovirus, *EBV* Epstein - Barr virus, *PCR* Polymerase Chain Reaction, *Anti-VGKC* Anti-voltage gated potassium channel, *MPO & PR3 ANCA* Myeloperoxidase and proteinase-3 anti-neutrophil cytoplasmic antibody, *C3 and C4* Complement component 3 and 4, *ANA* Anti-nuclear antibody, *Anti-CCP* Anti-cyclic citrullinated peptide, *Anti-RNP* Anti-ribonucleoprotein, *Anti-Scl 70* Anti-scleroderma, also known as anti-topoisomerase 1, *Anti-Jo-1* Antibodies to histidyl tRNA synthetase, *LDH* Lactate dehydrogenase, *CA-125* Cancer antigen-125, *β-HCG* Beta human chorionic gonadotropin, *AFP* Alpha-fetoprotein, *ANCA* Anti-neutrophil cytoplasmic antigen, *H. influenzae Haemophilus influenzae*, *S. pneumonia Streptococcus pneumoniae*, *N. meningitidis* Neisseria meningitidis, *HSV* Human simplex virus, *VZV* Varicella zoster virus

A week later, the clinical phenotype had evolved considerably from that of pure meningitis, to meningoencephalitis. On representing to the Emergency Department, she had additional clinical features of confusion with impaired concentration, memory deficits and mild dysarthria. She remained febrile. She rapidly became increasingly agitated, and developed visual hallucinations and a generalised tonic-clonic seizure. Over 5 days, she developed a reduced conscious level alternating with agitation and mutism. She was intubated and ventilated, and treated empirically with intravenous ceftriaxone 2 g a day and acyclovir 10 mg/kg three times a day for presumed meningoencephalitis. She was subsequently transferred to our hospital for further investigations and care. During this period, despite ongoing antimicrobial therapy for a week, she had ongoing pyrexia and a persistently reduced Glasgow Coma Scale (GCS) requiring critical care support. She later developed marked autonomic instability, along with new orofacial and upper limb dyskinetic movements.

She underwent detailed investigations for infective and autoimmune causes of encephalitis and meningitis. Her Magnetic Resonance Imaging (MRI) brain with contrast showed a few tiny non-specific supratentorial white matter T2-weighted high signal foci which were not thought to be of clinical relevance. Otherwise the appearance of the brain parenchyma was normal with no abnormal enhancement post-contrast. A summary of her serum and CSF investigations is shown in Table 1. CSF repeatedly demonstrated marked lymphocytosis with elevated protein levels, along with an elevated opening pressure on one occasion and a reduced CSF/serum glucose ratio on two occasions. Investigations for a number of infections were negative. An electroencephalogram showed generalized brain wave slowing indicative of diffuse cerebral dysfunction. Serum live cell-based assay for NMDA-receptor (NMDAR) antibodies from day 14 of illness was positive. CT of the pelvis demonstrated a left ovarian lesion. Subsequent excision confirmed a stage 1A G1 immature ovarian teratoma on histopathological examination. A diagnosis of anti-NMDAR encephalitis related to ovarian teratoma was made. Due to a sampling error, a CSF NMDAR antibody result was not available.

She was treated with high-dose intravenous methylprednisolone 1 g a day for 5 days on day 28 of illness followed by five cycles of plasma exchange, and subsequently started on prednisolone at a dose of 60 mg once daily via a nasogastric tube. She was extubated after 3 weeks and she continued to demonstrate improvement in her concentration, memory and language ability. After leaving intensive care, neuropsychiatric assessment documented executive dysfunction with concrete thinking and anxiety, however these rapidly improved. She underwent a left salpingo-oophorectomy 2 months following her initial presentation. She was discharged from hospital after 3 months at which point she was independent in her mobility and self-care. Steroids were slowly reduced and stopped over 3 months as an outpatient. Further immunotherapy was not required. Because of the clear clinical diagnosis, supported by immunological assay and clinical improvement, repeat CSF tests were not clinically indicated.

Overall, she made an excellent recovery. She has remained well and returned to work as a receptionist. Further assessments revealed only subclinical executive dysfunction with no major impact on her activities of daily living. Serial gynaecological oncology reviews confirmed complete excision of teratoma and absence of contralateral disease. Oncological prognosis is considered to be good. We consider the likelihood of recurrence of encephalitis to be low.

## Discussion and conclusion

We made a definite diagnosis of anti-NMDAR encephalitis associated with ovarian teratoma, despite an initially atypical presentation with a clinical syndrome of meningitis.

Differential diagnoses include a range of infective and autoimmune causes of meningoencephalitis. Following an initial illness phase of meningitis with raised CSF pressure, and prior to availability of antibody results, her clinical phenotype evolved to meet suggested criteria for “probable” anti-NMDAR encephalitis [1]. The subsequent development of memory deficits, psychiatric disturbances and seizures in the second phase of her illness is consistent with established clinical features of this condition [2, 3]. Rapid clinical progression to reduced consciousness alternating with agitation, development of autonomic instability and movement disorder is also well-described. Definite anti-NMDAR encephalitis was subsequently confirmed by a positive serum NMDAR antibody live cell-based assay.

Low-grade fever and non-specific headaches have frequently been reported in the prodromal phase of anti-NMDAR encephalitis. By contrast, signs of raised intracranial pressure including raised CSF opening pressures and bilateral abducens palsies have infrequently been reported. Also, very rarely patients with nuchal rigidity have been described [4]. Our case illustrates a rare set of presenting feature of NMDAR encephalitis with a prodrome of florid meningitis manifesting with nuchal rigidity, fever and elevated CSF opening pressure, lymphocytosis and raised protein.

The mechanisms underlying our patient’s presentation are incompletely understood. One question is whether this could represent post-infectious antibody generation, as is well-recognised several weeks after HSV encephalitis [5]. However, the very short time interval to emergence of fulminant encephalitis is against the possibility of de novo autoantibody generation [6]. Alternatively, a meningitis might induce transient blood-brain barrier disruption allowing the infiltration of NMDAR-specific B cells [2, 3]. If so, the patient should have pre-formed circulating NMDAR-specific B cells, and perhaps this is reflected by the finding of Dahm and colleagues who reported an unexpectedly high seroprevalence of N-methyl-D-aspartate-receptor subunit-NR1 autoantibodies in patients and healthy individuals [7]. Another possible mechanism could be that the meningitis directly activated CNS-surveying NMDAR-reactive B cells. This process would necessitate loss of immunological tolerance to this molecule and could account for the relative rarity of meningitis as a presenting complaint of anti-NMDAR encephalitis.

**Table:** 

We carried out a literature search in MEDLINE and EMBASE databases for original reports of anti-NMDA receptor encephalitis presenting with meningitis (defined as the presence of nuchal rigidity and photophobia in association with a headache) in humans, using the search criteria ‘anti-NMDA encephalitis’. A search between January 1990 and September 2017 yielded 384 potential articles. Screening of titles and abstracts led to exclusion of 307 articles. We reviewed the full text of the remaining 77. None described meningitis as a presenting feature. A cited reference search (Google Scholar) however identified one report describing meningitis as a presenting feature [4].	

This case highlights meningitis as an atypical presenting feature of anti-NMDA-receptor encephalitis. Clinicians should consider autoimmune encephalitides in individuals with meningitis, particularly where extensive investigations fail to identify a causative pathogen and there is rapid development of an encephalitic phenotype. A multidisciplinary approach is required to address the neurological, gynaecological, oncological, and neuropsychiatric aspects of this challenging and incompletely understood disorder.

## Data Availability

Not applicable.

## Abbreviations

Anti-NMDAR: Anti-N-methyl-D-aspartate receptor
CSF: Cerebrospinal fluid
CT: Computed Tomography
GCS: Glasgow Coma Scale
MRI: Magnetic Resonance Imaging
PCR: Polymerase chain reaction

## References

[1] Graus F, Titulaer MJ, Balu R (2016). A clinical approach to diagnosis of autoimmune encephalitis. Lancet Neurol.

[2] Irani SR, Bera K, Waters P (2010). N-methyl-D-aspartate antibody encephalitis: temporal progression of clinical and para-clinical observations in a predominantly non-paraneoplastic disorder of both sexes. Brain..

[3] Dalmau J, Gleichman AJ, Hughes EG (2008). Anti-NMDA-receptor encephalitis: case series and analysis of the effects of antibodies. Lancet Neurol.

[4] Kittichanteera S, Apiwattanakul M (2014). Meningitis as early manifestation of anti-NMDAR encephalitis. Neurol Asia.

[5] Bektaş Ö, Tanyel T, Kocabaş BA (2014). Anti-N-methyl-D-aspartate receptor encephalitis that developed after herpes encephalitis: a case report and literature review. Neuropediatrics..

[6] Nosadini M, Mohammad SS, Corazza F (2017). Herpes simplex virus-induced anti-N-methyl-d-aspartate receptor encephalitis: a systematic literature review with analysis of 43 cases. Dev Med Child Neurol.

[7] Dahm L, Ott C, Steiner J (2014). Seroprevalence of autoantibodies against brain antigens in health and disease. Ann Neurol.

